# Frequency of early postoperative adverse events (AEs) in adult patients undergoing elective neurosurgical intervention at tertiary care center in Pakistan

**DOI:** 10.12669/pjms.37.4.3501

**Published:** 2021

**Authors:** Mansoor Chandio, Faraz Shafiq, Syed Ather Enam

**Affiliations:** 1 Dr. Mansoor Chandio, FCPS. The Department of Anaesthesiology, The Aga Khan University, Stadium Road, Karachi, Pakistan; 2 Dr. Faraz Shafiq FCPS. The Department of Anaesthesiology, The Aga Khan University, Stadium Road, Karachi, Pakistan; 3 Dr. Syed Ather Enam, MD, FRCS, PhD The Department of Neurosurgery, The Aga Khan University, Stadium Road, Karachi, Pakistan

**Keywords:** Neurosurgical procedures, Post-operative, Pakistan

## Abstract

**Objective:**

The postoperative period is critical in neurosurgical patients, where the incidence of postoperative AEs is significantly high. Most of events occurs during recovery phase and has got relation to anaesthetic management. The objective of study was to determine frequency of early AEs in elective neurosurgical patients.

**Methods:**

This cross sectional study was conducted at our tertiary care center. The duration of study was one year, from August 2017-July 2018. The data was collected using predesigned proforma. The assessment was done on arrival in recovery room (T1) and then at forty five minutes (T2), twenty-fourth hour (T3) and forty-eighth hour (T4) postoperatively.

**Results:**

Total ninety-five patients were included. Overall, five hundred and forty AEs were recorded at T1, T2, T3 and T4. Anaesthesia related events like pain, postoperative sore throat, hoarseness, shivering and hypothermia were the commonest (73%). There was a gradual decline in incidence of these events over period of 48 hours. There was no effect of age, sex, BMI and blood loss on incidence of AEs.

**Conclusions:**

Postoperative pain, PONV and shivering were frequently reported AEs. We did not identify the impact of age, sex, BMI, comorbid or type of surgery in terms of having these events.

## INTRODUCTION

The postoperative period is critical in neurosurgical patients, demands individualized and procedural specific care. The incidence of postoperative adverse events (AEs) in this patient population is higher as compared to other surgical procedures.^1^ The reported incidence is up to 24 %.^2^ Most of the time these events occurs during immediate recovery phase. This relates not only to stress related to surgery, but anaesthetic intervention also plays major role in predicting short and long-term outcomes.^3^ However, association of AEs definitely worsens the outcome of neurosurgical patients and has direct impact on overall mortality.^4^ The early detection, rapid intervention and standardized care is key to successful management.^5^ This is not possible without having local data. In low middle-income countries (LMICs) where the resources are limited, the situation must be different. It is very difficult to determine the exact burden of these complications. The objective of this study was to determine the frequency of early AEs in elective neurosurgical patients at our tertiary care center.

## METHODS

This cross sectional study was conducted at post anaesthesia care unit and neurosurgical ward, at Aga Khan University Hospital Karachi. The duration of study was one year, from August 2017 - July 2018. The study protocol was approved from Ethical review committee of Aga University Hospital on 16^th^ January 2017 bearing letter no 4409-Ane-ERC-16, and informed consent was taken from patients fulfilling inclusion criteria. Non-probability consecutive sampling technique was used. All adult patients with age ranges between 18 to 70 years, undergoing elective neurosurgical procedures were included. The procedures were divided into either *Cranial or Spinal surgery*. Cranial surgeries included were elective tumor craniotomies. While procedures like laminectomy, discectomy, pedicle screw fixation and excision of spinal tumours were included as spinal surgical procedures. Patients already planned for postoperative intensive care unit (ICU) stay, had intraoperative major catastrophic event like cardiac arrest, myocardial infarction, major hypoxia and massive bleeding were excluded from study. Patients required unplanned ICU admission were also excluded from study protocol. The enrollment of patient into the study was started after hospital admission. The intraoperative anaesthetic care, monitoring, induction technique and maintenance of anaesthesia were at the discretion of primary anesthetist. After extubation, patients were shifted to PACU. An independent data collector was responsible for retrieving the data from patient progress notes and recording it on predesigned data collection form. All patients were reviewed immediately at the time of arrival in PACU (T1). The relevant clinical data was recorded from anaesthesia monitoring form. This included details about demographic data, anaesthetic care and surgical procedure. The patient was then reassessed at 45 minutes (T2) for any complications occurred at PACU. The recovery room chart was also reviewed. In ward, all patients were followed up at 24^th^ hour (T3) and then; the final assessment was done at 48^th^ hour (T4) postoperatively. The file notes and daily progress sheets were reviewed for any AEs that occurred during ward stay. However, in case of any ambiguity in documentation, relevant teams were contacted to overcome any bias. For all time intervals that is; T1, T2, T3 and T4, legends were marked for any events, even if it already been marked for previous time interval. Recorded AEs were categorized as:

### Anaesthesia Related

Airway trauma, postoperative pain, postoperative nausea/vomiting (PONV), postoperative sore throat (POST), shivering, and hypothermia.

### Surgery Related

Position related injuries, seizures, reoperation, Cerebrospinal fluid (CSF) leak, drop in GCS, new onset of neurological deficit.

### Cardiovascular

Hypertension (HTN), hypotension, arrhythmias, myocardial infarction (MI).

### Pulmonary

Desaturation, re-intubation, hypoventilation, pulmonary edema, acute respiratory distress syndrome (ARDS), respiratory failure, pneumonia and pulmonary embolism (PE).

### Metabolic

Diabetes Insipidis (DI).

Following definitions were used to mark and log the AEs.

### 1. Postoperative pain/post craniotomy pain

Procedural pain, the assessment of which was done using Numeric Pain Rating scale (NPRS) of 1-10.^6^

### 2. PONV

As assessed on the PONV impact scale.^7^

### 3. Airway trauma

Any trauma to Lips, teeth or tongue associated with intubation, it also includes hoarsness of voice/laryngeal edema

### 4. POST

Postoperative pain in throat related in intubation, with no preoperative history of such pain before the operation. It was assessed using verbal rating scale of Yes or No.

### 5. Hypothermia

Core body temperature less than 36 degree Celsius.

### 6. Shivering

Involuntary movement associated with hypothermia. It was assessed using verbal rating scale of Yes or No.

### 7. CSF leak

Evident CSF leakage from nose or in the posterior pharynx as complained by patient and confirmed by surgical team.

### 8. Position related injuries

Pressure sores, burns injuries related to positioning during surgery.

### 9. Seizures

Any seizure activity occurred during postoperative period. It was using verbal rating scale of Yes or No.

### 10. Reoperation

Re-exploration required with in a period of 48 hours after procedure.

### 11. New onset of Neurological deficit

Any cranial nerve, sensory or motor deficit.

### 12. Acute Drop in GCS

Sudden drop in GCS level for base line.

### 13. DI

Absolute or relative deficiency of Antidiuretic hormone postoperatively. This needs to be associated with increased urine volume and rise in serum sodium level. The diagnosis was made on the basis of clinical notes.

### 14. Pulmonary embolism

Symptomatic thromboembolic phenomena after surgery diagnosed after relevant investigations.

### 15. Hypertension

Systolic BP higher than 140 mmHg and diastolic higher than 90 mmHg.

### 16. Hypotension

Systolic BP less than 90 mmHg and diastolic less than 60mmHg.

### 17. Arrhythmias

Abnormal electrical rhythm of heart.

### 18. MI

Rise in serum Troponin levels with or without EKG evidence. This needs to be diagnosed by cardiology and documented accordingly.

### 19. Desaturation

Persistent drop in oxygen saturation (SPO_2_) less than 92% requiring supplemental oxygen.

### 20. Need for re-intubation

The patient needs securing of airway because of neurological status i.e. GCS < 8/15 or any other cause.

### 21. Hypoventilation

Respiratory rate of 8 breaths per minute.

### 22. Pulmonary edema

Abnormal accumulation of fluid in the extravascular compartments of the lung because of cardiac reasons.

### 23. Respiratory Failure

The term ‘respiratory failure’ is used when pulmonary gas exchange fails to maintain normal arterial oxygen and carbon dioxide levels.

### 24. ARDS

According to Berlins definition as follows.

**Table:** 

Timing	Within 1 week of a known clinical insult or new/worsening respiratory symptoms.
Chest imaging (CT Scan)	Bilateral opacities not fully explained by effusions. Lobar/lung collapse or nodules.
Origin of oedema	Respiratory failure not fully explained by cardiac failure or fluid overload.
	Needs objective assessment (e.g. echocardiography) to exclude hydrostatic oedema if no risk factor present.
Oxygenation	Mild—26.6 kPa < PaO2/FIO2PaO2/FIO2 ≤ 39.9 kPa with PEEP or CPAP ≥ 5 cm H_2_0
	Moderate—13.3 kPa < PaO2/FIO2PaO2/FIO2 ≤ 26.6 kPa with PEEP or CPAP ≥ 5 cm H_2_0.
	Severe—PaO2/FIO2PaO2/FIO2≤ 13.3 kPa with PEEP ≥ 5 cm H_2_O

### 25. Bronchospasm

Characterized by a critical limitation of expiratory flow.

### Statistical Analysis

Data was analyzed using statistical packages for social science version 19 (SPSS Inc., Chicago, IL). Frequency and percentage was computed for qualitative observations like gender, comorbid conditions like diabetes mellitus (DM), hypertension (HTN), ischemic heart disease (IHD), Asthma, and perioperative complications. Mean and standard deviation was estimated for age, height, weight, BMI, duration of surgical procedure and estimated blood loss. The frequency and percentages were also calculated for AEs at T1, T2, T3 and T4. Stratification analysis was done to evaluate the relationship between age, gender, BMI, comorbid, type of procedure and blood loos with the frequency of AEs. Chi-square test was used to observe difference. P≤0.05 was considered as significant. The sample size was calculated on the basis of previous reported incidence of Post operative complications in neurosurgical patients; that is 54.5,^8^ with 10% margin of error and 95% confidence interval.

## RESULTS

**Fig.1:**
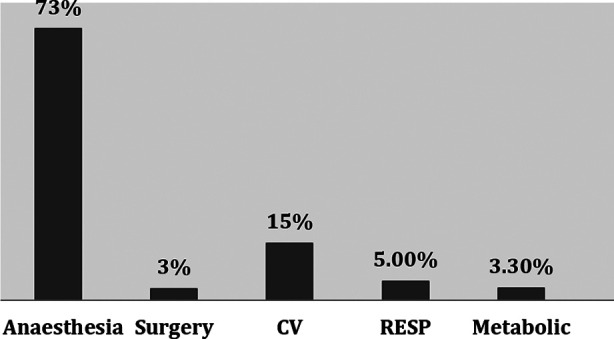
Overall frequency of AEs in neurosurgical patients.

**Fig.2:**
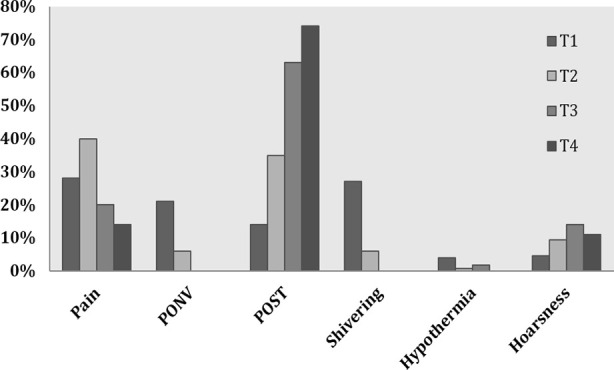
Comparison of anaesthesia related AEs at various time intervals

**Fig.3:**
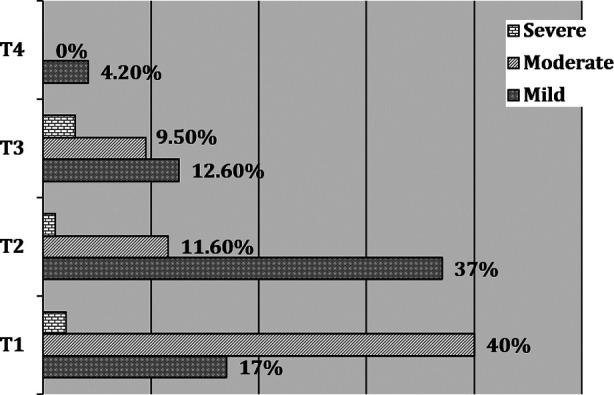
Severity of Pain at various time interval.

Demographic characteristics of patients are shown in Table-I. The preoperative health status was measured using American Society of Anesthesiologists (ASA) status. 14.74% of the participants had ASA status I, 62.11% had ASA status II and 23.16% had ASA status III respectively. HTN was the common comorbid condition (32.6%) followed by DM (11.6%), IHD (2.1%), and Asthma (2.1%). Amongst surgical procedures 42 patients (44.2%) had spinal, and 53(56%) had cranial surgeries. The mean duration of surgical procedure was 240.39±118.08 minutes. This was associated with mean blood loss of 486.68±454.88ml amongst our study participants. 14 patients were identified to have any intraoperative events (Table-II). Overall, 540 AEs were recorded at mentioned point intervals (T1, T2, T3 and T4). Amongst those, anaesthesia related events were at the top of the list (73%). This was followed by cardiovascular, respiratory, surgery and metabolic related events (Fig.1). There was a gradual decline in incidence of these events over a period of 48 hours, having peak at T1, while T4 was the time where the incidence was minimum (Table-III). The reported anaesthesia related events were pain, POST, hoarseness, shivering and hypothermia. At arrival in recovery room (T1), complain of having pain was at the top (25%), followed by postoperative shivering (22%). At 12^th^ hour (T2), pain and POST (30% and 25%) were the most frequent complications. While, POST was the main problem at 24^th^ (T3) and 48^th^ (T4) hour postoperatively (51% and 60%). (Fig.2). The severity of pain at various times interval is shown in Fig.3. Most patients had mild to moderate pain as measured on VAS.

**Table-I:** Demographic characteristics of study patients.

Variables	Point estimation
Age (years)	43.04±14.29
BMI (Kg/m^2^)	26.64±6.28
Duration of surgical procedure (minutes)	240.39±118.08
Blood Loss (ml)	486.68±454.88
Male	(65.26%)
Female	(34.74%)
***ASA Status***	
I	(14.74%)
II	(62.11%)
III	(23.16%)
***Comorbid Conditions***	
Hypertension	(32.6%)
Diabetic Mellitus	(11.6%)
IHD	(2.1%)
Asthma	(2.1%)
Others	(3.2%)

**Table-II:** Perioperative information about study patients.

Variables	Percentage %
***Surgical Procedure***	
Spinal	44.2%
Cranial	55.8%
***Surgical Position***	
Prone	36.8%
Supine	57.9%
Park Bench	2.1%
Lateral	3.2%
***Invasive Monitoring***	
Arterial Line	66.3%
Central Venous Line	23.2%
Any Intraopertive Event ( n=14)	**14.7%**
Blood Loss (6)	42%
Hemodynamic variation (6)	42%
Difficult Intubation (1)	7%
Different Bag mask ventilation (1)	7%

**Table-III:** Frequencies of AEs at various time intervals.

Total Events (n=540)	Overall (n)	T1 %	T2%	T3%	T4%
Anaesthesia related	397	49%	29%	15%	7%
Surgery related	16	37%	31%	19%	13%
Cardiovascular	83	55.4%	34%	6%	5%
Respiratory	26	85%	14%	4%	0%
Metabolic	18	33%	33%	33%	0%

Stratification analysis found no difference of age, sex, BMI and blood loss on incidence of AEs. Similarly, we didn’t identify any significant difference of these events in comparison to spinal versus cranial surgeries. However, metabolic events were more common in spinal surgeries (P= 0.073). Secondly, the pain was more in surgeries related to spine (P=0.040).

## DISCUSSION

The postoperative period in our study showed significant association with AEs. The anaesthesia related events were at the top of the list. This corresponds to the results of various studies, which showed immedaite postoperative period as the high risk for such events.^9^ This requires vigilant monitoring, frequent assessments and standardized care of these patients. In contarst to various reports, we did not indentifed the effect of ASA status, age and comorbid conditions on reported complications.^10^ This must be related to the fact that most of the events study were directly related to anaesthesia and can affect any one irrespetive of base line health status. Similarly, we did not identify any difference in relation to cranial or spinal surgeries. However, metabolic complications like DI and SIADH were more common in patients having cranial surgeries. Frequency of anaesthesia related AEs need to be focused here. At the time of arrival in PACU pain, shivering and PONV (28%:27%:21%) were most frequently reported.

At T2, pain and POST (40%:35%) were the most common. The same pattern was followed at intervals of T3 and T4, where the incidence of POST was 63% and 74% respectively. Most of our patients had mild to moderate pain as assessed using NPRS. However, we are not following any set protocol for postoperative pain management which could be the reason of this reporting. Studies have shown better pain management with the implementation of standarized protocol using patient controlled analgesia (PCIA).^11^

The overall incidence of PONV is bit low. This must be because of double antiemetic prophylaxis with Dexamethasone and Ondansteron, which we usually follow for our neurosurgical patients. The efficacy of regime is proven through various studies^12^ and the results here is validating the same. HTN and desaturation were the most frequent cardiorespiratory events recorded in this study. Most of these events were occur at T1 and T2. However, none of these patients required intuabtion or unplanned ICU admission. Though studies have shown residual neuromuscular blockage as the cause of respiratory compliations. The reported incidence is between 2 to 35. Postoperative HTN in our study could be directly related to pain or postopertaive shivering. We think that targeting both would resolve the issue related to HTN.

In contrast to results of various studies reporting hypotension during recovery phase,^13^ we didn’t observe this in our study population. Only seven patients had hypotensive episode at T1. The events related to surgery were very low. This could be directly related to the facts that patients here were elective, and well optimized. Only two patients reported dropped in GCS in PACU. Similarly only one patient had seizure episode postoperatively. The very low incidence of seizures in our cohort is reinforcing the efficacy of prophylactice use of Levetiracetam.^14^ Its routine for us to prescribe seizure prophylaxis after surgery. There are certain limitations associated with this study. First, the data is from elective neurosurgical patient only, that may not be the true reflection. Moreover, we didn’t followed the patients beyond 48 hours which might have missed the long term outcomes. Secondly, the data is from single centre which may not reflect the staus nationwide.

## CONCLUSIONS

In our elective neurosurgical patients, postoperative pain, nausea, vomiting and shivering were the frequently reported AEs. All of anaesthesia related events were peak at immediate recovery phase (T1 and T2) and got setteled with the passage of time (T3 and T4). We did not identify the impact of age, sex, BMI and comorbid conditions in terms of having these complications. Similarly, there was no significant difference in comparision to occurrence of these events in cranial versus spinal procedures.

### Authors’ Contribution:

**MC** conceived, designed and did statistical analysis. Also responsible and accountable for the accuracy and integrity of work.

**MC, FS** did data collection and manuscript writing and editing of manuscript.

**SA** did review and manuscript writing and design of study.
